# The association between the biomarker D-dimer and thrombosis in vein segment 5 among patients with first episode of deep vein thrombosis

**DOI:** 10.1016/j.clinsp.2026.101110

**Published:** 2026-09-05

**Authors:** Izzet Altintas, Baker Nawfal Jawad, Nora Olsen El Caidi, Sanaá Elmajdoubi, Thomas Kallemose, Gencay Gül, Beth H. Olsen, Nikolaj Eldrup, Kasper Karmark Iversen, Lene Juel Rasmussen, Anette Sjøl, Jan O. Nehlin, Ove Andersen

**Affiliations:** a Department of Clinical Research, Copenhagen University Hospital Amager and Hvidovre, Hvidovre, Denmark; b Emergency Department, Copenhagen University Hospital Amager and Hvidovre, Hvidovre, Denmark; c Ultrasound Department, Copenhagen University Hospital Amager and Hvidovre, Hvidovre, Denmark; d Department of Vascular Surgery, Copenhagen University Hospital Rigshospitalet, Copenhagen, Denmark; e Emergency Department, Copenhagen University Hospital Herlev and Gentofte, Herlev, Denmark; f Center for Healthy Aging, Department of Cellular and Molecular Medicine, University of Copenhagen, Denmark; g Department of Cardiology, Copenhagen University Hospital Amager and Hvidovre, Hvidovre, Denmark; h Department of Clinical Medicine, University of Copenhagen, Denmark

**Keywords:** Biomarkers, D-Dimer, Deep vein thrombosis, Vein segment 5

## Abstract

**Objective:**

Precise anatomical localization of lower extremity Deep Vein Thrombosis (DVT) has been advocated to improve diagnostic characterization and risk stratification beyond the traditional proximal-distal classification. The objective of this study was to examine whether first-episode DVT involving segment 5 veins differs from non-segment 5 DVT with respect to d-dimer levels and clinical characteristics.

**Method:**

In this retrospective observational study at Copenhagen University Hospital-Amager and Hvidovre, Denmark, from March 1st, 2017 to February 28th, 2019, we included patients with first-episode lower extremity DVT admitted to the Emergency Department. Patients were divided into two groups: segment 5 vein thrombosis and non-segment 5 vein thrombosis. Differences in d-dimer levels between the groups were analyzed using unadjusted and adjusted linear regressions models with d-dimer as the dependent variable.

**Results:**

Of 150 patients with a first-episode lower extremity DVT, 147 had available d-dimer measurements. d-dimer levels were significantly higher in patients with segment 5 vein thrombosis than with Non-segment 5 vein thrombosis. In the unadjusted analysis, mean d-dimer levels were 5.2 Fibrinogen Equivalent Units higher (CI: 2.2:9.8, p = 0.012). After adjustment, this difference increased to 6.5 Fibrinogen Equivalent Units (CI: 1.0:13.0, p = 0.041). These findings suggest that Segment 5 vein thrombosis is associated with increased thrombus degradation and a higher inflammatory burden. This may have significant implications for future risk stratification and the clinical management of DVT.

**Conclusion:**

First-episode Segment 5 vein thrombosis was associated with significantly higher d-dimer levels than Non-segment 5 vein thrombosis

## Highlights

• Segment 5 vein thrombosis carries higher risk of adverse outcomes.
• D-dimer levels are higher in Segment 5 vein thrombosis vs. other segments.
• Segment-specific DVT classification may improve thrombus characterization.

## Introduction

Deep Vein Thrombosis (DVT) and Pulmonary Embolism (PE) collectively make up Venous Thromboembolism (VTE), a potentially life-threatening condition characterized by blood clot formation.

In 2016, the Society for Vascular Surgery (SVS) and the American Venous Forum (AVF) emphasized the need for improved precision in the diagnosis of DVT, and proposed a standardized anatomical classification of six predefined anatomical segments to improve the precision and consistency of DVT characterization.^1^ Rather than categorizing DVT as “proximal” or “distal” this framework specifies thrombosis according to individual vein segments and anatomical location.^1^ This distinction enables more refined assessment of thrombosis and may have clinical relevance, as thrombosis in more proximal segments has been associated with elevated risk of long-term complications.^1^^,^^2^

De Maeseneer et al. (2016).^1^ followed SVS and AVF recommendations to describe thrombus extent by subdividing the extremity into five segments. However, the study, did not investigate any associations between biomarkers and thrombosis within specific vein segments.^1^ A subsequent study by Broholm et al. (2019), used the classification from De Maeseneer et al. (2016) to assess the anatomical distribution of acute DVT,^3^ but did not investigate biomarker differences across vein segments.

While previous studies have applied the segment-based classification to describe anatomical distribution, the biological implications of thrombus localization remain insufficiently explored. d-dimer was first recognized as a useful marker for DVT in the 1980s. Its role as a predictor of DVT emerged from studies showing that elevated d-dimer levels were associated with thrombotic events.^4^ d-dimer is a specific fragment resulting from crosslinked fibrin degradation product,^5^ and can rule out DVT suspicion, in patients with a low to moderate pre-test probability of the condition.^6^ Research has shown that elevated d-dimer levels are indicative of the presence of a thrombus in lower extremity.^7^ To the best of our knowledge, the association between d-dimer levels among patients with Segment 5 vein thrombosis and those with Non-segment 5 vein thrombosis has not previously been reported.

This single-centre retrospective study aimed to investigate the association between patients with Segment 5 vein thrombosis compared to Non-segment 5 vein thrombosis and d-dimer levels upon admission to the Emergency Department (ED), among patients with first-episode lower extremity DVT, given the nomenclature disagreement noted above. In the present study, the vein segments were classified as follows: Segment 1 includes the calf veins, which consist of the anterior and posterior tibial veins, the peroneal vein, and the muscular veins, such as the gastrocnemius or soleus veins. Segment 2 corresponds to the popliteal vein, while Segment 3 encompasses the femoral vein. Segment 4 is defined as the common femoral vein. Segment 5 includes the external iliac vein and the common iliac vein, which may be accompanied by the inferior vena cava. These divisions align with the classifications recommended in the 2016 guidelines set forth by the Society of Vascular Surgery and the American Venous Forum. Building upon these prior recommendations, the present study also introduces an additional segment, Segment 6: the vena profunda femoris is proposed as a distinct category. Its unique anatomical location and potential clinical relevance justify a separate classification within the venous segmentation system.

By integrating anatomical vein segmentation with biomarker assessment, this study aims to clarify whether Segment 5 thrombosis is associated with increased d-dimer levels in patients with first-episode lower extremity DVT.

## Methods

### Setting

This study is a single-center, retrospective observational study performed at the ED, Copenhagen University Hospital Amager and Hvidovre, Hvidovre, Denmark. This study utilized 20.400 electronic patient records from patients admitted at the ED with various symptoms over a course of 24-months, from 1st March 2017 to 28th February 2019.

### Population

Patients were eligible for inclusion in the study if they were ≥18-years of age and had their first episode DVT in a lower extremity confirmed by compression ultrasonography performed by expert staff from the Department of Radiology, Copenhagen University Hospital Amager and Hvidovre. Patients with DVT referred to the ED primarily came from the primary care sector, although the study also included those who were admitted to inpatient beds or received outpatient care at the hospital.

### Data extraction

Data was extracted from the Electronic Healthcare Records (EHR) by study staff and transferred to a research database in the Research Electronic Data Capture program (REDCap). Study staff developed a manual with a priori definitions to ensure uniform data entry. After completing data entry into REDCap, a manually quality control of biomarker variables was performed.

### Clinical examination and diagnostic procedures in the ED

Patients suspected with DVT upon admission underwent a clinical probability test with Wells score,^8^ and a d-dimer measurement was performed to either support or rule out the suspicion of DVT.^9^ A panel of experts in the field of radiology performed compression ultrasonography with doppler effect to confirm or rule out the DVT diagnose.

The compression ultrasound examination was performed on the site of the extremity with symptoms. Subsequently, the vessels of the limbs were visualized by compression ultrasonography in supine or prone position by radiologists at the Department of Radiology, Hvidovre Hospital, to confirm or disprove the initial DVT diagnosis in the lower extremity. Compression ultrasonography in B-mode imaging was performed from ankle to groin to assess vein compressibility. The absence of Doppler flow with or without color, and any discomfort related to the transducer (pain) was examined. Loss of vein compressibility was used as the sole criterion for diagnosing DVT.

For optimal visualization, high-frequency (4‒10 MHz) linear array transducers were preferred. The narrow, flat footprint of the linear array facilitated the application of direct and even pressure to the vessel(s) of interest, during the examination. Larger, lower-frequency curvilinear probes were used for obese, pregnant or patients with reduced insights in the vessels requiring deeper visualization when necessary.

In our study, prior to data extraction and processing, we adopted a scoring system in which each thrombosed vein segment identified by compression ultrasonography was assigned 1-point. This score was later used to calculate the thrombus score.

### Variables

The following description is related to variables that are standard clinical procedures, measurements, and biomarkers recorded as part of usual care for patients with DVT in the ED, Copenhagen University Hospital, Amager and Hvidovre. No measurements or procedures specific to this study were conducted.

### Clinical signs upon admission at ED

Upon patient admission, National Early Warning Score (NEWS) (based on systolic blood pressure (mmHg), heart rate (beats/min), respiratory rate (breaths/min) and oxygen-saturation (%) were assessed as DVT symptoms, and objectively confirmed clinical signs, including lower extremity laterality, were assessed using the Wells score.

### Measurement of biomarkers

As part of standard care, patients with DVT admitted to the ED had blood samples collected within the first two hours of hospitalization, including d-dimer which was measured by IL d-Dimer HS HemosIL. Physicians retrieved biomarker values from the clinical laboratory information system, LABKA, and transferred to the REDCap database. The measured d-dimer levels were adjusted using age-specific reference thresholds.

Oxygen saturation (%) was measured using a pulse oximeter and the probe with the sensor was attached to the index finger.

### Localization of vein segments and thrombus score

**Fig. 1:**
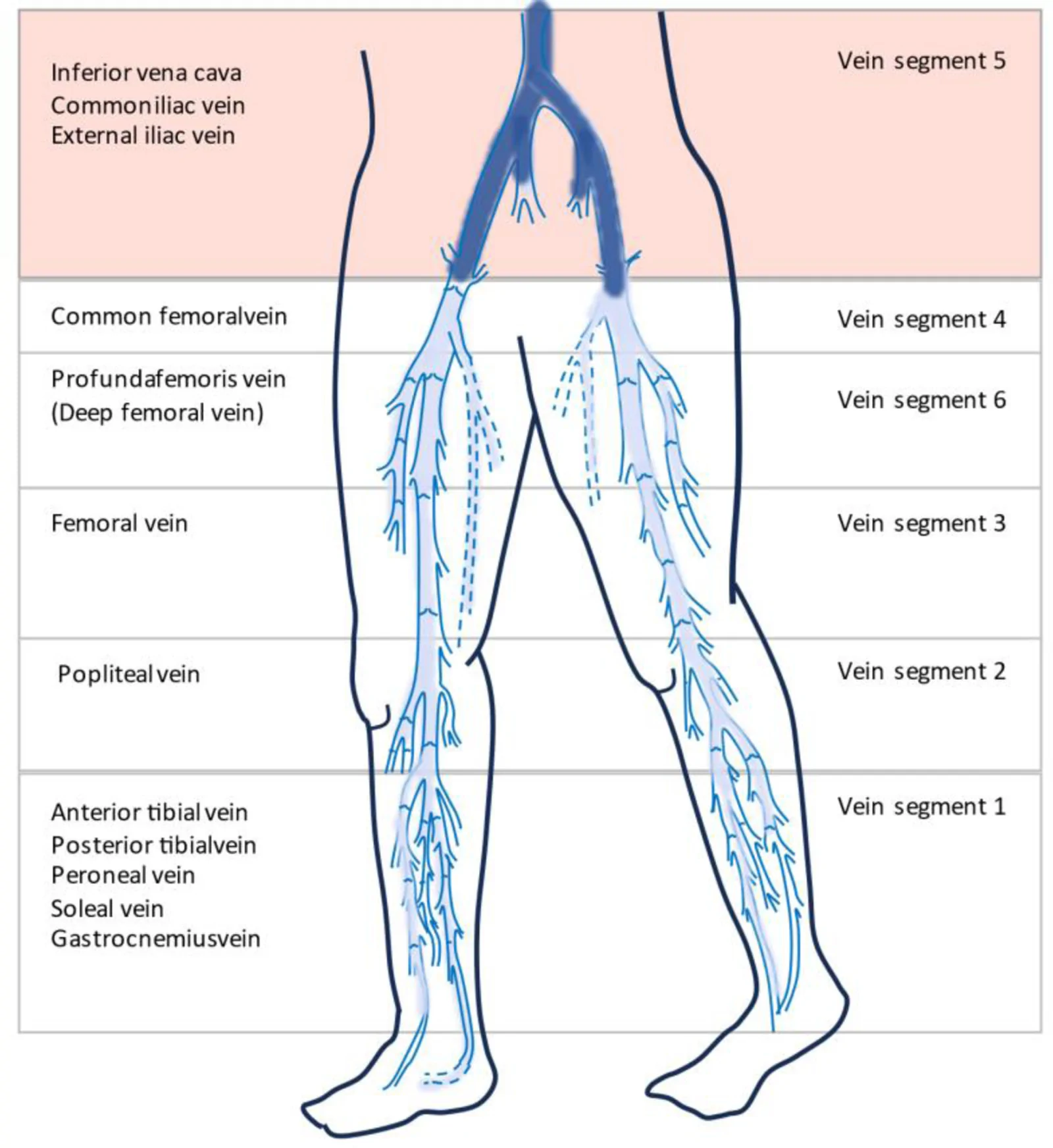
Schematic illustration of lower extremity vein segments used for DVT assessment by compression ultrasonography. The venous vasculature is divided into vein Segments 1‒6.

Lower extremity vein segments were defined a priori and applied consistently during compression ultrasonography and subsequent data extraction (Fig. 1).

Although anticoagulant therapy was indicated irrespective of thrombus localization, Segment 5 vein thrombosis may in selected cases prompt consideration of thrombolytic intervention. Although anticoagulant therapy was indicated irrespective of thrombus localization, Segment 5 vein thrombosis may in selected cases prompt consideration of thrombolytic intervention.^10^ Patients were therefore grouped according to the presence of Segment 5 vein thrombosis versus thrombosis in all other vein segments, collectively referred to as Non-segment 5 vein thrombosis. Thrombosis in Segments 1, 2, 3, 4, and 6 (Non-segment 5 vein thrombosis),^11^ was likewise recorded and included in subsequent analyses described in the following sections.

### Outcomes and covariates

The primary outcome was the baseline level of the biomarker d-dimer, expressed in Fibrinogen Equivalent Units (FEU). Covariates included grouping variables for Segment 5 vein thrombosis and non-segment 5 vein thrombosis, along with age, sex, and individual variables indicating the presence of thrombosis in vein Segments 1, 2, 3, 4, or 6.

This study was conducted following the STROBE statement

### Statistical analysis

Continuous variables are presented as medians with Interquartile Range (IQR), due to skewed distributions, while categorical variables are presented as numbers with percentages. Analysis comparing d-dimer levels between patients with Segment 5 vein thrombosis and patients with Non-segment 5 vein thrombosis were done using liniar regression models, with d-dimer levels as dependent variable. Both unadjusted models, which included only Segment 5 vein thrombosis or Non-segment 5 vein thrombosis variable as independent variables, and adjusted models, which additionally included the remaining covariates, were fitted. Model assumptions were evaluated by QQ-plots and fitted vs. residual value plots. Since deviations from model assumptions were observed, 95% Confidence Intervals (95% CI) and p-values were estimated using the bootstrap method with 10.000 bootstrap samples. d-dimer levels were missing for three patients, multiple imputation using chain equations, 5 imputations based on age and sex were performed. Pooled analysis using the imputed datasets were done as an sensitivity analysis, these analysis used the same liniar regression models as the original analysis; p-values <0.05 were considered statistically significant. All analyses were conducted using *R* version 4.1.0 (R Foundation for Statistical Computing, Vienna, Austria).

## Results

**Fig. 2:**
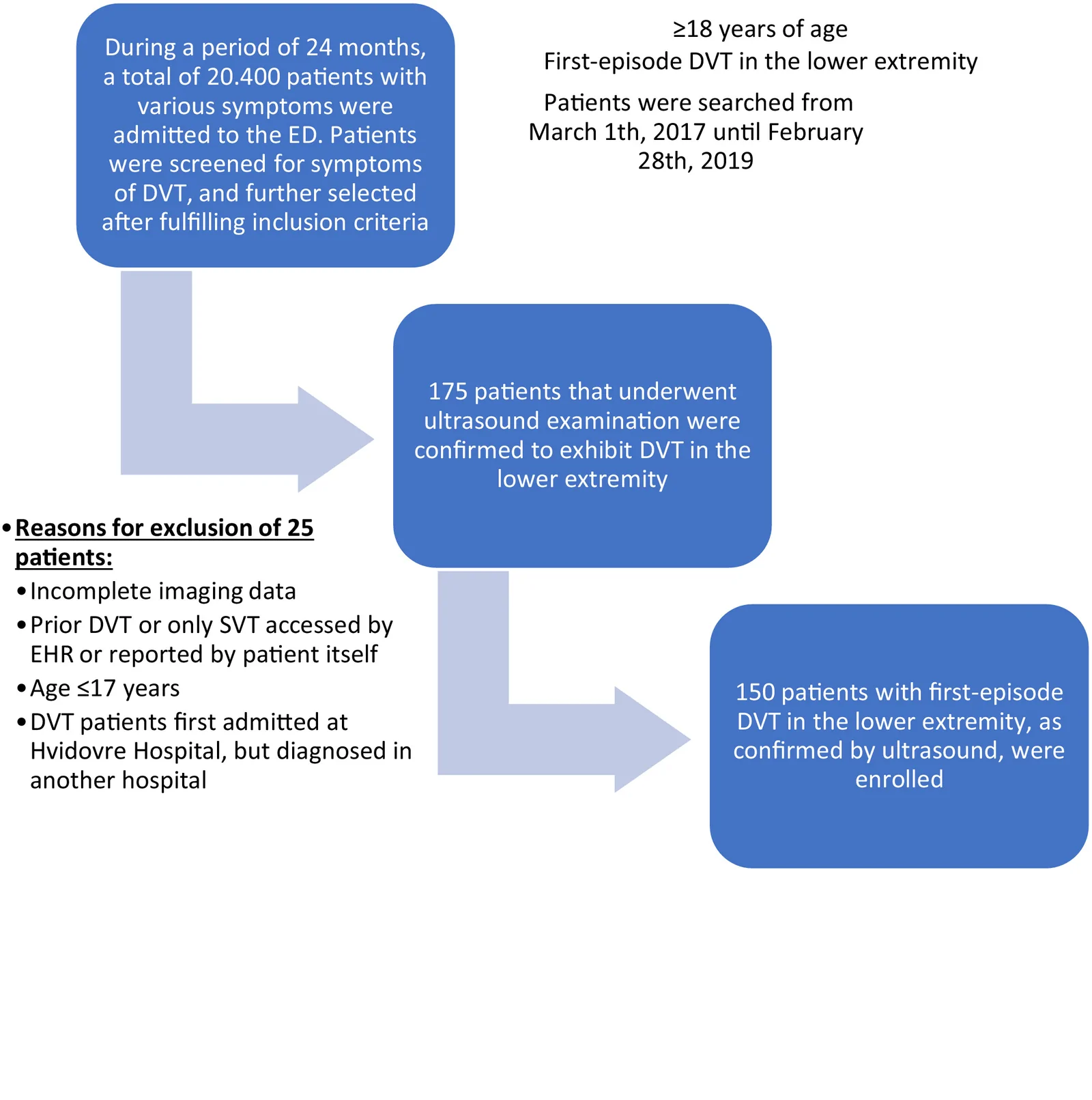
Flowchart illustrating the participant selection for the study, including patients experiencing their first-episode of acute lower extremity DVT. Patients were admitted at ED between March 1st, 2017, and 28th February 2019. After the exclusion procedure, a total of 150 patients with first-episode lower extremity DVT were diagnosed by compression ultrasonography and included in the analysis. DVT, Deep Vein Thrombosis; ED, Emergency Department; HER, Electronic Healthcare Record; SVT, Superficial Vein Thrombosis.

In total, 20.400 patients with various symptoms were admitted to the ED. After screening for DVT symptoms, those meeting the inclusion criteria were selected. Among them, 175 patients were diagnosed with lower extremity DVT using Compression Ultrasonography (CUS). After excluding patients due to incomplete imaging data, a history of prior DVT, only superficial vein thrombosis, or diagnosis of DVT at another clinical site despite admission to Hvidovre Hospital, a total of 150 patients with first-episode lower extremity DVT were included in the analysis. The inclusion and exclusion criteria are shown in Fig. 2 below.

Table 1 presents the characteristics of the patients included in the study cohort. The median age was 57.0 years (IQR: 46‒71). A total of 23 patients (15%) had Segment 5 vein thrombosis, whereas 127 patients had non-segment 5 vein thrombosis.

**Table 1:** Baseline clinical characteristics for patients experiencing their first episode of lower extremity deep vein thrombosis.

**Baseline clinical characteristics**	**Total patients with DVT**	**Segment 5 vein thrombosis**	**Non-segment 5 vein thrombosis**
Patients with DVT, n (%)	150 (100%)	23 (15%)	127 (85%)
Age (Years)	57 (46:71)	67 (33:81)	57 (47:68)
Sex (n Female)	64 (43%)	12 (52%)	52 (41%)
D-dimer (FEU)	2.2 (1.0:5.6)	5.6 (2.5:12)	1.8 (0.9:4.7)
NEWS			
Systolic blood pressure (mmHg)	135 (124:150)	130 (122:144)	137 (125:150)
Diastolic blood pressure (mmHg)	80 (72:90)	77 (70:85)	82 (73:90)
Heart rate (beats/min)	80 (72:90)	84 (77:92)	80 (70:90)
Respiratory frequency (breaths/min)	16 (16:18)	18 (16:20)	16 (16:18)
Temperature ( °C)	36.6 (36:37)	36.5 (36:37)	36.6 (36:37)
Saturation (% Oxygen)	98 (96:98)	96 (95:98)	98 (97:98)
Clinical assessment			
Wells score	2 (1:3)	2 (1:3)	2 (1:3)
Thrombus score	2 (1:3)	3 (3:4)	1 (1:2)
Pregnancy^a^ (n)	21 (33%)	5 (42%)	16 (31%)
1.Trimester (n)	15 (23%)	2 (17%)	13 (25%)
2.Trimester (n)	1 (2%)	1 (8%)	0 (0%)
3.Trimester (n)	5 (8%)	2 (17%)	3 (6%)
Laterality			
Dxt (n)	65 (43%)	6 (26%)	59 (46%)
Sin (n)	83 (55%)	16 (70%)	67 (53%)
Bilateral (n)	2 (1%)	1 (4%)	1 (1%)
Thrombolysis treatment (n)	17 (11%)	17 (74%)	0 (0%)
24 months mortality (n)	8 (6%)	4 (17%)	4 (3%)

Data presented as numbers and percentages (%) or as median with Interquartile Range (IQR).

a Percentage based on number of females. DVT, Deep Vein Thrombosis; Dxt, Dexter; FEU, Fibrinogen Equivalent Unit; NEWS, National Early Warning Score; Sin, Sinister.

Patients with Segment 5 vein thrombosis showed a higher thrombus score compared to patients with Non-segment 5 vein thrombosis (median 3 [IQR: 3‒4] vs. median 1 [IQR: 1–2]). The distribution of sex, National Early Warning Score (NEWS), Wells score, pregnancy trimester overview, and laterality is shown in Table 1. Only patients with Segment 5 vein thrombosis received thrombolysis (74%). There was a higher 2-year mortality rate for patients with Segment 5 vein thrombosis compared with patients with Non-segment 5 vein thrombosis (17%vs. 3%).

The median d-dimer FEU was 5.6 (IQR: 2.5‒12.0) in patients with Segment 5 vein thrombosis and 1.80 (IQR: 0.9‒4.7) in patients with Non-segment 5 vein thrombosis (Table 1). The analysis showed patients with Segment 5 vein thrombosis had significantly elevated levels of d-dimer compared to those with Non-segment 5 vein thrombosis, with an average difference of 5.2 FEU (CI: 2.2:9.8, p = 0.012) for the unadjusted analysis and 6.5 (CI: 1.0:13.0, p = 0.041) in the adjusted analysis. Sensitivity analysis using multiple imputation to account for missing values showed similar results and did not lead to different interpretations of the results.

**Fig. 3:**
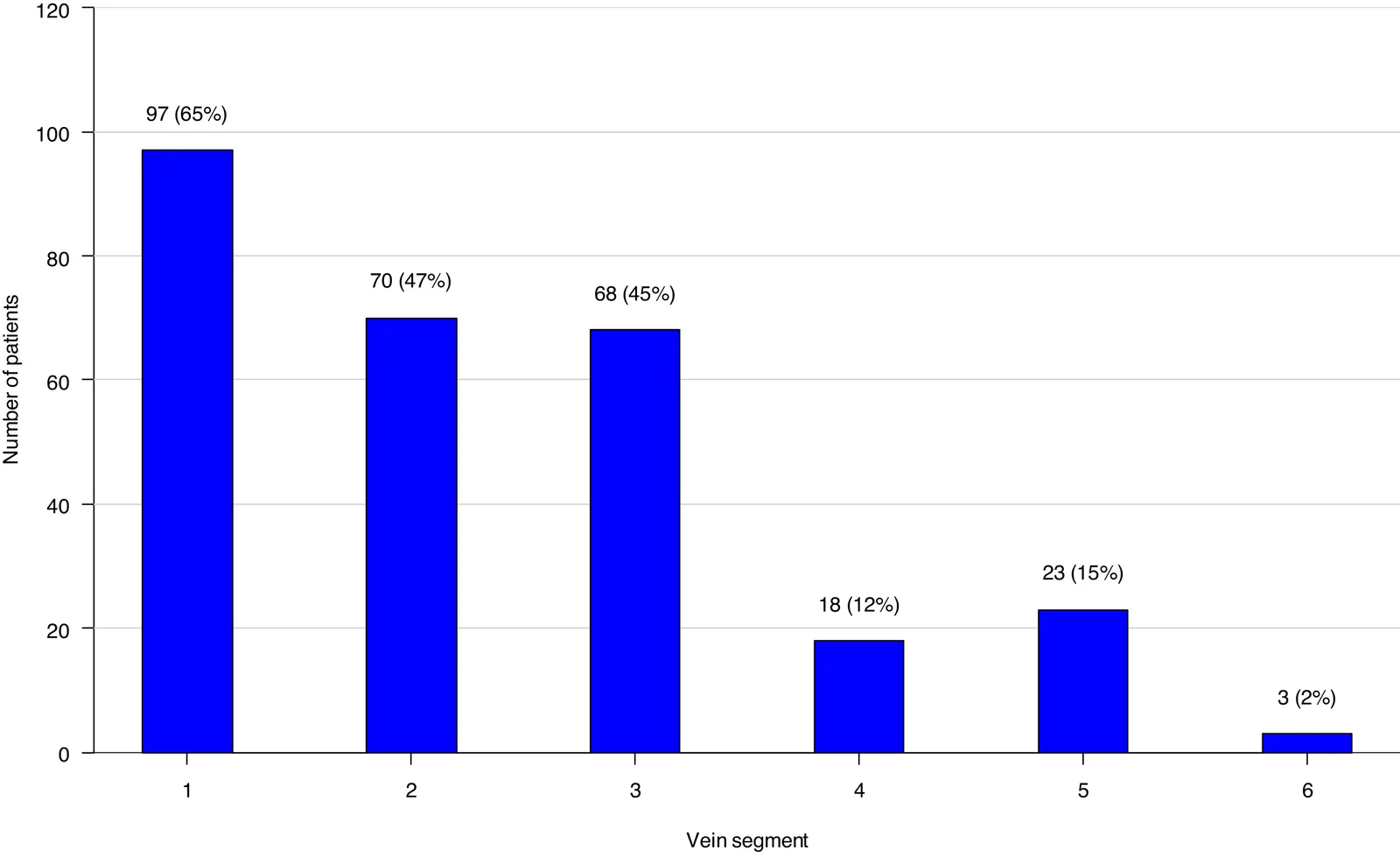
Thrombus location in DVT patients according to vein Segments 1‒6. Diagram illustrating the thrombus location according to vein Segments 1‒6, with a detailed description of the subdivision of the vein segments in lower extremity, including the anatomical names of the veins. Vein Segment 1: Calf veins: V. tibialis anterior and posterior, peroneal vein, muscular veins: v. gastrocnemius or v. soleus. Segment 2: Popliteal vein. Segment 3: Femoral vein. Segment 4: Common femoral vein. Segment 5: External iliac vein, common iliac vein with or without Inferior vena cava. Vein Segment 6: Vena profunda femoris.

The majority of patients had thrombosis located in vein Segment 1 (Fig. 3). The distribution of thrombus location in the lower extremity is presented as counts (N) and percentages (%) according to the respective vein segments (Fig. 3).

## Discussion

In this retrospective observational, single-center cohort study, we identified an association between Segment 5 vein thrombosis and elevated d-dimer levels at admission among patients experiencing their first episode of lower extremity DVT. This finding is of particular clinical importance as Segment 5 vein thrombosis is associated with an increased risk of the long-term complication post-thrombotic syndrome PTS,^12^ as well as a higher mortality.^13^

Recent evidence shows that inflammation contributes to DVT formation.^14^^,^^15^ The association of thrombosis in specific vein segments with elevated inflammatory biomarkers could potentially impact the management and treatment of patients with DVT, allowing healthcare professionals to tailor treatment plans based on the patients' inflammatory profiles. Patients with thrombosis in the external iliac vein, common iliac vein, or the inferior vena cava (vein Segment 5) have an increased risk of developing PTS, compared to those with thrombosis in other lower extremity veins.^16^, ^17^, ^18^, ^19^, ^20^

Previous studies have also demonstrated that d-dimer plasma levels correlate with the anatomical extent or severity of acute symptomatic DVT in the lower limbs.^7^^,^^21^ Also, it was found that d-dimer levels correlated with the extent of DVT, with small calf thrombi being associated with low d-dimer levels.^22^ However, that study^22^ did not investigate whether this correlation was applicable across different vein segments. The observed association in the present study suggests that these patients may be at higher risk of: a) Elevated activity of the coagulation cascade, accompanied by increased fibrinolysis activity,^23^ b) Higher thrombotic burden^24^ and increased coagulability,^25^ c) Higher inflammation levels,^26^^,^^27^ and d) Recurrent DVT may have an increased risk of developing PTS^17^^,^^28^ compared with patients with non-Segment 5 vein thrombosis.

The thrombus score reflects the thrombus burden in the lower extremity.^29^ The 3-fold higher median thrombus score among patients with involvement of Segment 5 vein thrombosis (who often also had thrombosis in other segments) may indicate a more pronounced overall thrombus burden, with patients potentially requiring longer anti-coagulant treatment and having a higher risk of residual thrombosis after completing treatment compared with patients with Non-segment 5 vein thrombosis.

Previous studies have shown that the risk of developing PE^30^ and mortality^31^^,^^32^ is lower in patients with calf DVT compared to those with ilio-femoral or proximal vein thrombosis, which is associated with significantly higher all-cause mortality rates.^33^ This underscores the critical role that the anatomical location of the thrombus plays in determining patient outcomes.

Accurately studying thrombosis in certain veins, presents several challenges. For instance, De Maeseneer et al.^6^ noted in 2016 that they had to exclude the internal iliac vein (included in Segment 5) and the profunda femoris vein (Segment 6) from their study due to missing data. This limitation highlights a gap in our understanding and risk assessment of thrombosis in these specific vein segments.

In response to these challenges and to improve risk stratification, we included and evaluated thrombosis in all specific vein segments as recommended by The Society for Vascular Surgery and The American Venous Forum,^34^ including thrombosis occurring in vein Segments 6 (Vena profunda femoris). Importantly, we placed particular emphasis on vein Segment 5 due to its clinical significance. Segment 5 vein thrombosis is associated with a higher risk of complications and often necessitates advanced treatments in selected patients.^10^ Focusing on this critical vein segment enhances risk prediction and provides a more precise understanding of how thrombosis location influences long-term patient outcomes.

### Strengths

The strengths of our study are as follows: Firstly, only patients with first-episode lower extremity DVT were included in the study, therefore, the measured d-dimer in these patients is believed to be unaffected by prior DVT episodes. Secondly, a panel of experts from the Department of Radiology conducted the CUS and were not involved in the clinical examination or treatment of the patients. Thirdly, the study deals with real-world data, providing results that span across demographics in a real-world setting, thereby avoiding selection bias.

### Limitations

Our study has limitations that warrant consideration. Firstly, the Danish population is predominantly Caucasian, limiting generalizability of our findings to other ethnicities. Notably, hospital discharge data from California in 1996^35^ revealed significantly higher VTE events among African-American compared to Caucasians. Unfortunately, due to the demographics of our study population, we lack data for comparison with other ethnic groups such as African Americans, Asians, and Hispanics. Secondly, the limited number of patients with Segment 5 vein thrombosis likely contributed to the wide confidence intervals of the estimates. Consequently, while an association between Segment 5 vein thrombosis and d-dimer levels was identified, the magnitude of this association and its clinical relevance remain difficult to determine. Thirdly, all patients with Segment 5 vein thrombosis also had thrombosis in other segments, which made it more difficult to isolate the independent effect of Segment 5 from the remaining segments, as these likely also contribute to increase the d-dimer level. However, we attempted to address this limitation by including the presence of thrombosis in each vein segment as separate variables in the adjusted analysis to isolate segment specific effects. Fourthly, we did not further investigate the presence of concomitant PE in patients, as a prior study reported silent PE in one-third of patients with DVT.^36^ This remains an important consideration, since the measured biomarker, d-dimer, may be further elevated due to endogenous fibrinolysis in DVT patients who also experience a PE event. For the fifth point, we would also like to clarify that the findings may reflect clot size or thrombus extent, rather than a unique property of Segment 5 giving the small sample number of patients with first-episode of Segment 5 vein thrombosis.

## Conclusion

In conclusion, first-episode Segment 5 vein thrombosis was associated with substantially higher d-dimer levels compared with thrombosis in other vein segments. Although the subgroup size was modest, the magnitude of the difference suggests a higher thrombotic burden in this anatomical location. Larger studies are needed to confirm these findings and refine the estimate of the effect size.

## Perspectives of the findings

An important unmet requirement in DVT research is the identification and validation of biomarkers that can reliably, accurately, and rapidly predict long-term clinical outcomes, such as reduced thrombus resolution, recurrent thrombosis, and the development of PTS. These complications often lead to patient re-admissions to the ED, needing re-evaluation by clinicians and further blood biomarker level measurements in laboratory panels. When biomarkers capable of predicting long-term complications in patients with DVT are identified at baseline, this knowledge could represent a significant step forward, enabling clinicians to use additional tools to improve DVT treatment from the time of diagnosis. Elevated inflammation levels and reduced fibrinolytic capacity may contribute to initial DVT occurrences.

In a prospective study, we are measuring levels of various inflammatory biomarkers, including the chronic inflammation biomarker suPAR, senescent cells, immune cell counts and their characteristics, as well as systemic plasma profiles. Our aim is to assess whether plasma and cellular biomarkers can help predict long-term complications, such as PTS, from the time of initial DVT diagnosis (ClinicalTrials.gov Identifier: NCT05789108 and VEK nr: H-22,011,783).

## Agreement to share publication-related data and data sharing statement

Data underlying the findings of the study are available from the corresponding author upon reasonable request. Data sharing is subject to relevant approvals and a formal data transfer agreement in accordance with Danish data protection legislation and institutional policies (izzet.altintas@regionh.dk).

## Ethics

The study was conducted in accordance with the Declaration of Helsinki and complied with Danish legislation.

This study is based on data extraction from patient records, following internal review and approval as a quality assurance project by the hospital management of Copenhagen University Hospital, Amager and Hvidovre (Workzone: 8500,299), including access to the medical records. The aim of the quality assurance study was the investigation of the quality of diagnosis and treatment of patients with DVT in the ED at Hvidovre Hospital, as well as whether referrals for thrombolysis intervention are made according to current clinical guidelines. Informed consent for participation was not applicable.

## Declaration of generative AI in scientific writing

AI-assisted technology has not been used in the preparation of this work, except for grammar and spelling checks.

## Authors’ contributions

Authors IA, NE and OA were involved in the study design. IA, BNJ, NO, SA, TK were involved in data collection, data interpretation and data analysis. IA, BNJ, OA and JN drafted the manuscript, while all other authors performed critical review and provided revisions. All authors approved the final version of the manuscript.

## Funding

This research was funded by The Capital Region of Denmark. The study sponsors had no role in study design, collection, analysis, and interpretation of data, in writing the manuscript or decision to submit the manuscript for publication.

## Conflicts of interest

The authors declare no conflicts of interest.
